# COVID-19 rapid testing in a homeless shelter: Lessons for equitable access and sustainable pandemic response

**DOI:** 10.1016/j.ssmqr.2026.100754

**Published:** 2026-04-03

**Authors:** Rebecca Ziolkowski, Jillian Kelley, Graylin Skates, Lara Balian, Tiwaladeoluwa Adekunle, Natalia M. Rodriguez

**Affiliations:** a Department of Public Health, College of Health and Human Sciences, Purdue University, West Lafayette, IN, USA; b Department of Anthropology, College of Liberal Arts, Purdue University, West Lafayette, IN, USA; c Department of Obstetrics and Gynecology, University of Wisconsin-Madison, Madison, WI, USA

## Abstract

People experiencing homelessness (PEH) faced heightened vulnerability during the COVID-19 pandemic, yet little is known about how frontline homelessness service organizations navigated the public health responsibilities that shifted onto them as broader systems strained. This study provides a longitudinal qualitative analysis of on-site rapid COVID-19 testing implemented at a large Midwestern homeless shelter, examining how multi-level structural conditions shaped the feasibility, ethics, and sustainability of testing over the course of the pandemic.

Using semi-structured interviews with shelter guests (n = 42) and staff (n = 12) alongside document and policy timeline analyses (March 2020-January 2023), we conducted reflexive thematic analysis guided by a socioecological framework. Findings show that on-site rapid testing reduced longstanding barriers to care for PEH, offered reassurance in a high-risk environment, and enabled timely outbreak control. However, limited isolation capacity, rapidly evolving guidance, and the withdrawal of external resources generated significant operational and ethical challenges. As federal, state, and local supports diminished, responsibility for disease mitigation increasingly shifted onto the shelter, revealing the institutional precarity of social service agencies that were pushed into assuming public health responsibilities despite lacking the infrastructure to sustain them.

Ultimately, shelter leadership discontinued on-site rapid testing after determining that continued isolation and outbreak management were incompatible with the organization's mission and physical constraints. This study demonstrates that while rapid testing can advance health equity for structurally vulnerable populations, sustainability requires coordinated policy frameworks, dedicated resources, and recognition of the limits of frontline service organizations. These findings have implications for designing effective and equitable public health responses beyond COVID-19.

## 1. Introduction

People experiencing homelessness (PEH) were among the most vulnerable populations during the COVID-19 pandemic, as constrained access to housing, healthcare, and other social supports magnified both biological risk and social precarity. Higher rates of underlying medical conditions and exclusion from central public health directives to ‘stay at home’ and ‘maintain social distance’, meant PEH experienced disproportionate rates of infection, hospitalization, and death compared to the general population (Baggett, Keyes, et al., 2020; Chassman et al., 2023; Corey et al., 2022; Fenley, 2021). As other healthcare and community resources became restricted during the pandemic, homeless shelters became critical front-line institutions, simultaneously attempting to fulfill their core missions of rehousing and meeting basic survival needs, while also functioning as substitute healthcare access points for COVID-19 information and resources. Shelters were suddenly forced to implement disease control measures that were often at odds with their design and capacity, as congregate environments amplified the risk of transmission during a time of record increases in homelessness rates in the US. (Ghinai et al., 2020; Kaur et al., 2022; Levesque et al., 2022; Rodriguez et al., 2021)

Early outbreaks in large congregate shelters underscored how crowded conditions, limited resources, and inadequate infrastructure created ideal circumstances for rapid disease spread (Ghinai et al., 2020; Mosites et al., 2020). In response, the U.S. Department of Health and Human Services emphasized testing in congregate settings as an urgent protective measure for both PEH and shelter staff (U.S. Department of Health & Human Services, 2020). While homeless shelters attempted to implement infection-control strategies such as symptom screening, quarantine, and environmental modifications, they faced critical challenges including limited funding, staffing and supply shortages. Additionally, many of these measures, such as temperature and symptom checks, often proved insufficient on their own, due to the large numbers of asymptomatic cases that contributed to viral transmission. Testing for SARS-CoV-2 was rapidly promoted by federal agencies as a cornerstone of outbreak control, yet PEH faced major barriers to accessing community-based testing, including transportation, stigma, and limited healthcare resources (Aranda-Díaz et al., 2022; Mosites et al., 2020; Rodriguez et al., 2021, 2022a). Providing rapid testing within homeless shelters ultimately emerged as both a practical infection control strategy and, for many PEH, their only pathway to COVID-19 testing.

Initial studies highlighted the promise of shelter-based testing for identifying cases and reducing outbreaks, calling for broader implementation and expansion of isolation options. However, most early evaluations occurred during short windows of the pandemic, often in large cities with multi-agency partnerships and access to external facilities, resources not universally available across the country. As the pandemic persisted and testing supplies became more widely available through federal support in 2021, shelters began incorporating onsite testing into routine operations. Yet little is known about how testing supported or reshaped shelter operations, how staff and guests who relied on the testing navigated its consequences, and how resource and policy environments shaped their feasibility and sustainability.

To address disparities in COVID-19 testing access, the National Institutes of Health launched the Rapid Acceleration of Diagnostics–Underserved Populations (RADx-UP) program. RADx-UP funded community-engaged research to expand testing in populations disproportionately affected by the pandemic, including racial and ethnic minority groups, rural residents, and PEH. Its goals included not only increasing access to diagnostic testing but also generating evidence on how structural inequities shaped testing implementation and uptake in diverse, underserved contexts. Recent RADx-UP studies demonstrated the value of community partnerships and low-barrier testing models in reaching structurally vulnerable populations, such as co-locating testing in trusted community settings or embedding testing within service organizations (Aranda-Díaz et al., 2022; Ngandu et al., 2024; Rodriguez et al., 2024). Yet gaps remain in understanding how homeless shelters themselves experienced and navigated testing interventions over the course of the pandemic.

In order to better prepare for future public health crises, it is critical to understand how frontline organizations navigated the opportunities and challenges of integrating rapid testing into their daily operations and service provision. While previous studies have demonstrated the effectiveness of COVID-19 rapid testing in congregate settings, few have explored its organizational impact or the lived experiences of shelter staff and guests. This study, conducted as part of RADx-UP, addresses that gap by qualitatively examining the implementation of shelter-based COVID-19 rapid testing in a Midwestern homeless shelter, with the objective of understanding its role and impacts, both positive and negative, on shelter operations and the lived experience of shelter guests during the pandemic.

### 1.1. Study design and public involvement

This work was supported by the National Institutes of Health Rapid Acceleration of Diagnostics-Underserved Populations (RADx-UP) initiative, and is a Community-Based Participatory Research (CBPR) project building upon previous work with a large homelessness services organization in the Midwest (Rodriguez et al., 2021, 2022b, 2024; Ziolkowski et al., 2024). After the organization implemented voluntary COVID-19 rapid antigen testing in their shelter, questions of mutual interest to both community and academic partners emerged, guiding the development of our research question. Beyond this, the organization or members of the study population were not directly involved in the research process, however relevant findings were shared in real time with the organization to inform the shelter's COVID-19 response.

Our hypothesis was that rapid COVID-19 testing in a homeless shelter setting would provide important benefits to shelter operations, including rapid identification of cases and avoidance of larger outbreaks, but could also introduce challenges or disruptions to shelter workflow and daily activities of homeless guests. To address this, the study followed a descriptive qualitative design to obtain specific and straightforward accounts from staff and guests related to the implementation of shelter-based rapid testing without imposing an interpretive framework beyond the data.

#### 1.1.1. Research team

All study design was developed by the PI, and all onsite research including participant recruitment, informed consent, and data collection was managed and conducted by a certified community health worker (CHW). The CHW, though not a member of the study population, was a woman local resident trained and certified on human subjects research ethics and had partnered with the homelessness services organization for 2 years prior to the start of this project to assist with healthcare navigation, COVID-19 mitigation, and previous research efforts. During this time, the CHW established relationships with guests and staff, and was known as a resource for COVID-19 education and resources. CHW roles beyond research were seized before this study began and the refocus of the CHW's work to that of solely research was made clear to participants where relevant. Additional members of the research team who were familiar with the organization and/or supported project efforts were involved in the analysis of data. This study was reviewed and approved by Purdue University's institutional review board (IRB-2021-1147). The funder did not have any role in the design or conduct of the research.

### 1.2. Recruitment, consent, and interviews

Recruitment was passive, namely through a study flyer placed above the testing station at the shelter and included a 5-min rapid assessment survey to determine that testing was recent, happened at the shelter, and future contact preference if interested in participating in an interview. Any shelter guest who was at least 18 years of age and had recently taken a rapid test at the shelter was eligible to participate and informed of the opportunity and purpose of the interviews, which took place within a private space at the shelter approximately 1–3 weeks after testing. Shelter staff were invited to participate in a semi-structured interview of approximately 30 min. Staff members were eligible to participate if they were at least 18 years of age and worked at the shelter. Staff members were recruited through purposive sampling in order to include perspectives across the organizational levels. Informed written consent was obtained from all participants. Upon completion of the interview, all participants were compensated with a $25 gift card to a local grocery store.

The CHW read all consent materials aloud to participants so as not to assume literacy and for consistency between both groups. During the consent process, it was clearly explained that participation was entirely voluntary and that their relationship with the organization or any services received would in no way be affected by their participation in the study, and that they had the right to withdraw at any time. Interview questions were semi-structured and informed by our previous work (Ziolkowski et al., 2024). Examples include, “What problems, if any, did rapid COVID testing help solve for the shelter and its guests?” and “‘what problems, if any, did it create?“. All participants were informed during consent that interviews would be audio recorded to ensure accuracy and interviews were concluded when the research team determined thematic saturation was achieved.

### 1.3. Analysis

#### 1.3.1. Document analysis

A document analysis was conducted in order to better understand the role of COVID-testing in the shelter throughout the evolving state of pandemic response. In addition to tracking national, state, and county-level policies from publicly available websites (i.e., CDC, The National Governors Association, and the local County Health Department's archived press releases) (Association, 2020; CDC, 2023), we examined organizational policy related documents, such as flyers, referral forms, and emails provided by the shelter, as well as weekly meeting minutes from the Health and Wellness Committee (HAWC), a collective of academic researchers, providers, and homelessness service professionals (Rodriguez et al., 2024). Relevant data were extracted on topics pertaining to the shelter's COVID-19 response and policies during the pandemic from March 2020 to January 2023. Findings were sorted by key topics then added to an excel sheet where relevant points in time from the federal, state, county, and shelter COVID-19 responses were collected and built into a timeline (Fig. 2).

#### 1.3.2. Qualitative analysis

All interviews were transcribed using Otter.ai and reviewed for accuracy by a second member of the research team. Following Braun and Clarke's six phases of reflexive thematic analysis (Braun & Clarke, 2021), the team first familiarized themselves with the data and generated initial codes, and separate codebooks for staff and guest interviews were developed through an iterative process that combined deductive codes from interview guides with inductive coding additions throughout the coding process. Coding was conducted in Nvivo, with two research team members coding each transcript independently before meeting to resolve differences. Consensus was reached through discussion. Codes were then grouped and refined into candidate themes, which were reviewed to ensure they were coherent and distinct, and further defined and named for clarity. Once coding was complete, the team synthesized themes into overarching categories of benefits and challenges, which were then organized using the socioecological model to guide interpretation. Transcripts were revisited as needed throughout the entire process to ensure coding consistency.

### 1.4. Positionality and reflexivity statement

The first author's positionality as both an insider and outsider to the shelter community was actively examined throughout this study. As a CHW with experience engaging shelter guests and staff as a health resource since the early COVID-19 pandemic, she entered this research with knowledge of structural barriers, local community dynamics, and some personal histories of participants, yet does not share the lived experience of being unhoused. This established rapport and the years of trust built through service engagement were instrumental in eliciting more candid and nuanced accounts than might otherwise be shared with an unfamiliar researcher, among a population with significant reasons to distrust institutional research. At the same time, we recognized that this dual role could still influence participants responses or they may hesitate to share negative feedback regarding shelter operations.

To navigate the power shift from service provider to researcher, several deliberate strategies were used. Crucially, the first author transitioned out of her role as a CHW early in the study development process to maintain a clear boundary during data collection. During the informed consent process, she explicitly defined her role as a researcher and affirmed that participation would have no bearing on participants' access to services or their care relationship. Throughout data collection, reflexive journaling was used to critically process the transition from provider to learner. Throughout the analysis, reflexivity was emphasized given the research team's longstanding collaboration with the shelter. This included ongoing discussion of how our positions and relationships with the organization shaped interpretation, with team members regularly raising and responding to questions that emerged during analysis.

## 2. Results

### 2.1. Participant characteristics

We interviewed n = 42 PEH and n = 12 staff members of the organization from December 2021 to August 2022. All PEH participants were current guests of the shelter and had received at least one rapid test at the shelter. Of the 42 guests interviewed, 17% were being tested for the first time prior to the interview. Of the 14 staff interviewed, 64% held direct guest engagement roles, such as front desk, case management, and health director, and 36% held roles in shelter administration and leadership.

#### 2.1.1. Context and timeline

##### 2.1.1.1. The setting.

The homelessness services facility studied is a nonprofit organization located in the Midwest that functions as a coordinated entry point for those in a housing crisis. They serve approximately 2000 clients per year, which they refer to as “guests” and have the primary mission of ending homelessness through their Rapid ReHousing program but also make available a variety of other supportive services for PEH. Their facility is designed to be a communal space with dedicated offices for other local organizations, such as mental and physical healthcare providers, to be onsite and provide PEH with quicker access to services, support, and resources in one location. The facility also addresses basic needs through access to toiletries, showers, laundry, and three meals per day, seven days per week. These services are primarily accessed through a large common area, referred to as “the day room”, that is entered through the main entryway and monitored by a front check-in desk. Outlined with doors to offices and the basic resources available (laundry room, lockers, kitchen serving window, etc.) the room holds 20 long tables that function as a space for guests to wait to be met with by case managers and service providers, eat their meals and rest. On average, staff report serving approximately 80 guests during each mealtime and it is then where general announcements for those seeking services are made.

**Fig. 1.**
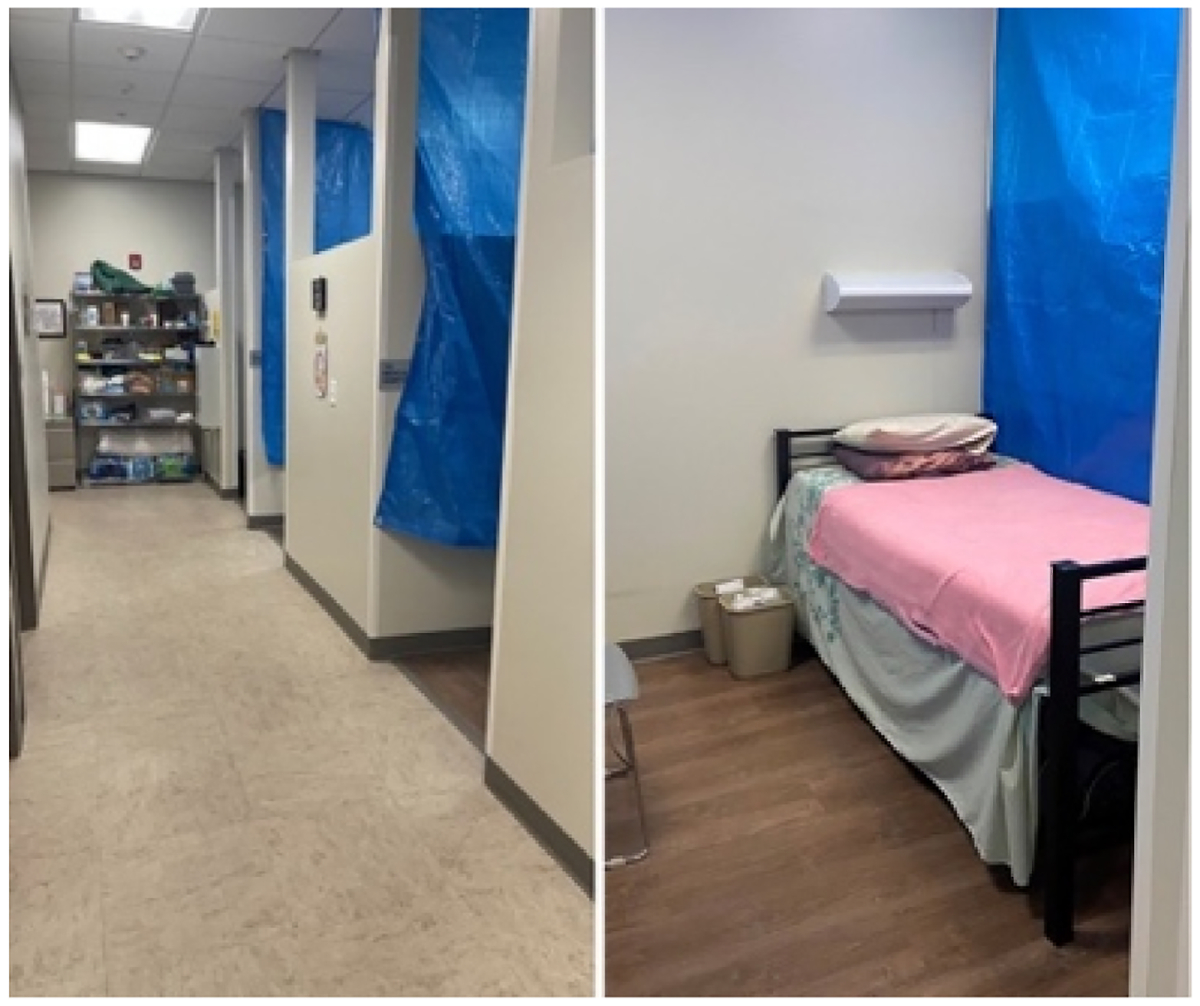
A photo depicting the shelter's medical respite pods that were adapted for COVID-19 by adding tarps for barriers over wall openings.

In addition, the facility has a medical respite area, which holds the clinic office space for visiting health service providers, the health director's office, and four beds for guests with health conditions requiring access to a daytime area to rest and recover. Rapid testing typically took place in the respite clinic, and the four respite bed areas were later utilized for isolating guests who tested positive for COVID-19 between March 2021-January 2023 (Fig. 1).

##### 2.1.1.2. Shelter COVID-19 testing procedures and timeline.

**Fig. 2.**
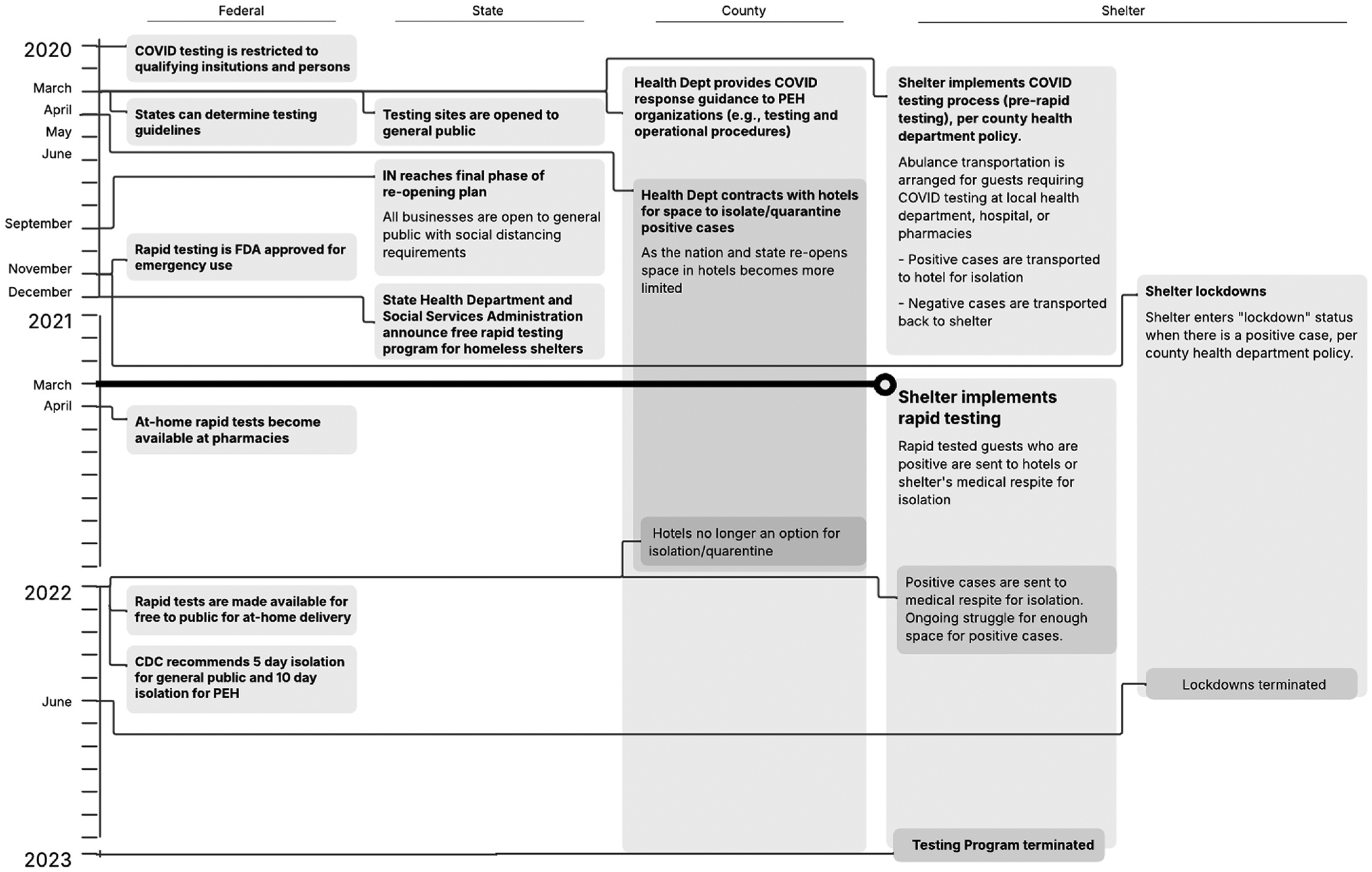
Timeline of COVID-19 testing procedures at the shelter, and relevant guidance and measures at the county, state, and federal levels.

Fig. 2 depicts a timeline of the homelessness services organization's process for COVID-19 testing and how it evolved from March 2020 to January 2023. As this process was influenced by larger social contexts that contributed to the impact of rapid testing on shelter operations, this timeline includes related guidance and measures at the county, state, and federal levels.

###### 2.1.1.2.1. Shelter procedures before onsite rapid testing.

The shelter testing procedures prior to having rapid testing available onsite were instructed by the local health department (HD) on March 23rd 2020, through broader guidance to encourage all local organizations serving PEH to stay open by modifying services and implementing measures to minimize community spread of COVID-19. Procedures outlined for the shelter by a HD brief included guidance for suspected cases:

“Call HD COVID-19 hotline if clients have symptoms and they will notify 911 if patient needs to be transferred to ER for testing/further evaluation. The patient will then be transported from hospital to isolation at a motel where HD nurses will monitor them and meet basic needs. If HD hotline staff determine the client doesn’t meet criteria to be tested, client will be placed in isolation (if possible at shelter) or HD will arrange for transport to the motel and HD will coordinate meals.”

Guests who tested negative at the hospital or other testing site were responsible for coordinating their own transportation back to the shelter, adding to logistical challenges for this population. As pandemic response resources and funding availability waned from 2020 to 2022, responsibilities such as sourcing and funding hotels for isolation were taken on by the shelter administration. Hotels became harder to source as the state reached its final phase of public reopening in September 2020, and the shelter eventually began to utilize their respite space as an isolation zone, first only when it was available, then converting it into predominantly an isolation zone as they saw increased waves of variants. This meant that guests needing respite space for other health reasons were often turned away.

###### 2.1.1.2.2. Shelter procedures upon implementation of onsite rapid testing.

In December 2020, the state department of health and social services administration announced a program to equip homeless shelters across the state with COVID-19 rapid testing capacity by working with shelters to acquire CLIA waivers and free rapid tests (BinaxNOW ^™^ cards) “*in order to rapidly identify positive cases to help with cohorting and contact tracing*”. Shelters were instructed, “*Testing materials are limited and should be used judiciously. Consider limiting use to symptomatic individuals and those that they have been in direct contact with*.” The shelter applied to the program and requested materials, which arrived for the first time in March 2021. After training CHWs on testing procedures and state reporting requirements, the shelter began onsite rapid COVID-19 testing on March 23, 2021.

Testing was voluntary for guests, however on occasion CHWs would approach those showing symptoms and encourage testing. If a guest or staff member requested to be tested, the CHW would take them to the designated testing space and conduct the procedure per the test manufacturer instructions. During the instructed 15-min wait-time, the CHW would wait with the patient and utilize this time to complete state reporting requirements. Results were then discussed with the patient and next steps were determined. If positive, guests were isolated in the medical respite space immediately after case identification for the CDC-recommended period at the time (14, 10, and eventually 5 days). If guests tested negative, they were advised to test again in the next few days.

##### 2.1.1.3. Lockdowns and isolation periods.

“Lockdowns” were an additional policy initiated and monitored by the HD in November 2020 to control the spread of COVID-19 by having the shelter not admit guests who were not at the facility within a week's time of the case discovery. When rapid testing began at the shelter, the increased accessibility of testing meant an increase in detection of cases and therefore more frequent lockdowns. The durations of lockdowns varied, starting at 14 days, and would be extended when new positive cases were identified. There were at least 4 documented lockdown periods (according to HAWC meeting minutes) with some extending over a month's time. In response to lockdowns during winter months, multiple community partner organizations came together to create temporary indoor spaces at a local organization and church, called the “annex”, for PEH unable to enter the facility because of the quarantine status. It was only open during some of the lockdowns and required a shelter referral for access. The lockdown policy was terminated in June 2022 through deliberation by the shelter and the HD of its benefit versus impact on providing shelter services and was determined no longer necessary especially with considerations of vaccine availability and uptake.

Isolation periods were also advised by the health department but as COVID-19 progressed and the shelter became more independently responsible for isolating guests in the respite area, the shelter health director would refer to CDC guidance and modify based on the shelter's experience with positive cases and ongoing outbreaks at the time, though they would always still inform and consult with the HD on these decisions.

#### 2.1.2. Benefits and challenges of on-site rapid testing

Participants (guests identified as G(x) and staff as S(x)) described a range of experiences with the implementation of rapid COVID-19 testing at the homeless shelter, revealing both the perceived value and the operational and ethical dilemmas that emerged. Thematic analysis identified key benefits and challenges across the individual, interpersonal, institutional, and community levels of the socioecological model.

##### 2.1.2.1. Individual-level experiences; improved access to testing vs. struggles created by shelter exclusion and isolation.

At the individual level, participants widely recognized on-site rapid testing as a practical and necessary response to structural barriers to care. Many shelter guests would have otherwise foregone testing due to limited mobility, financial constraints, or lack of information. One staff member explained, “They don't have easy mobility to get to a clinic, or they just don't know, you know. And that's a good thing that we were able to do that [rapid testing] here” (S9). The shelter environment was seen as particularly well-suited to rapid testing, with another staff participant noting, “Rapid testing was the only thing that could have worked within our world. And I think we wouldn't have gotten nearly, I mean; quarter of the people would have tested if we didn't have those right now” (S13). Avoiding external medical systems also alleviated cost burdens: “It makes it easier for people … to not have to go [to] the hospital and have that medical financial burden” (S6).

Access to rapid testing not only supported early detection, but also facilitated timely navigation to care, particularly for medically vulnerable populations. As one staff member put it, “It's been able to get people care that needed it that were positive, because we do have a lot of comorbidities here” (S13).

However, the benefits of rapid testing were at times overshadowed by the consequences of positive results. Lockdowns meant denying shelter access to new guests, even in times of urgent need. “Ever since COVID started, we've had to lock down anytime we had a positive here … it was 14 days in the beginning … That's a long time to be out in the cold if you're somebody waiting to try and get into a shelter” (S10). Another staff member echoed this concern, highlighting the moral tension of exclusion: “The struggle's really been when we had to quarantine [lockdown] and not let people in who were homeless in the winter. An’ even anytime, even in warm weather … that's been a real struggle when we're not available to people who need us” (S13).

Guests who were placed in isolation reported challenging and unhygienic conditions, and limited staff support. With only 1 or 2 staff being allocated to COVID-19 mitigation, their absence sometimes meant gaps in care, as one guest recounted, “It was like the 11th day, and I was like, cause um, ‘you wash my clothes, right?’ Cause I didn't have anything else to wear … I couldn't take a shower or nothing …” (G32). Participants also expressed concern about being exposed to additional health risks during isolation: “I sanitize my own room, I had to sanitize the other two guys' rooms too, because there was nobody [staff] there. I could have got sicker” (G32).

##### 2.1.2.2. Interpersonal dynamics; peace of mind and protection vs. stigma and mistrust from procedural breakdown.

Rapid testing also impacted interpersonal relationships within the shelter, fostering both reassurance and strain. For some staff and guests, the ability to quickly confirm negative status offered peace of mind. “It kind of eased that anxiety and it made it a little bit easier to work in an environment like this,” a staff member shared (S9). Guests expressed similar sentiments: “It made me feel better. That I know I don't got it. Because I had a feeling, well, I might have it, you know, not even know it” (G27). Guests also appreciated the ability to protect others at the shelter “to make sure the safety of myself and others around me” (G12). The testing process was also seen as addressing the broader emotional burden of living in a high-risk environment: “It can help solve some of the guests' anxiety about it. Sitting out there too—it's a high-risk environment” (S11).

However, isolation triggered negative emotional responses. One guest described the experience as monotonous and emotionally draining: “It's nuts—you just sit there and watch the walls” (G16). Concerns about privacy and stigma were also salient among those who tested positive. One guest recounted how testing lacked discretion: “And um [staff] was taking me back into that special room and everybody's looking. And they announced right in the midst of this everybody needs to start wearing their mask again. Somebody's been tested positive” (G103).

Communication breakdowns between staff and guests compounded mistrust. Some guests feared expulsion after a positive result. A staff member reflected, “A lot of people thought that if they had COVID, we were going to kick them out. So, there was a little bit of miseducation out there in regard to that. They didn't realize that we had a place that we could isolate them, if that did happen” (S10).

##### 2.1.2.3. Institutional impact and limitations; outbreak control and operational autonomy vs. moral and logistical challenges.

At the institutional level, rapid testing was seen as a critical infection control strategy. The immediacy of results allowed staff to isolate positive cases quickly, minimizing the risk of outbreaks in a densely populated shelter environment. “We could isolate them as quickly as possible, and hopefully not spread [COVID-19], as much. And so, then we could keep our doors open for people that needed our help to come in” (S3). Another staff member emphasized the advantage of on-site testing in limiting further exposures: “Because so many of them sleep in the same places. So, it's easier for us to contact trace … that it’s helped in that a lot. If you're waiting for like PCR tests or you're sending them somewhere else, you're just exposing more people …” (S6).

The operational flexibility enabled by rapid testing also facilitated daily shelter management. “Rapid testing helped us get to the point where we knew what we needed to do that day, you know, within that 15 min so we could start planning and it was just easier to set things into place” (S6). Another staff member noted how it contributed to organizational autonomy and less dependence on the local health department: “Since we have been able to do the testing … I think that just gives a sense of ownership over the process … to be able to make our decisions on our own” (S2).

Nonetheless, testing implementation surfaced tensions around enforcement and infrastructure. Staff tasked with enforcing lockdown protocols experienced moral discomfort. “Being on the desk, I actually hated when we had to go quarantine [lockdown], just for the simple fact that I'm the point person turning people away” (S3). Isolation capacity was severely limited, creating logistical challenges. “It caused issues with again, space … if we have more than four positives back in the respite because that's all the beds we have, where are we going to put people?” (S11). Another participant described the shelter's physical infrastructure as fundamentally inadequate: “First of all, we hope that it doesn't happen [more than 4 guests needing to isolate at the same time]. We say our Hail Mary's. We really don't have a good solution right now, because we have no areas in our building that we can put in any beds to go anywhere … it wasn't built for this. It's not set up for this” (S10). Participants also noted that identifying positive cases created a moral imperative to respond—without always having the means to do so. “Knowing they were positive, meant we needed to do something, and we didn't always have resources to care for them” (S13).

From the organizational leadership perspective, lockdowns also affected shelter intake metrics, generating concerns about perceived performance: “When we [locked] down, we [were] not allowed to enroll people. So, our numbers [were] going down [and] that [looked] bad on us” (S13). Policy dissemination within the organization was also perceived as inconsistent and ambiguous. One staff member described this dynamic: “I feel like the policies [are] a little bit iffy … I don't know that there's that great of a dissemination” (S8).

##### 2.1.2.4. Community-level perceptions; limiting broader transmission through early support vs. declining support and shifting burden of responsibility.

From a community perspective, rapid testing was seen as an effective strategy to limit broader transmission. The ability to test and isolate on-site reduced the need to send symptomatic guests into the public. As one staff member noted, “Instead of having to go to a hospital or off site to get tested to limit the risk of exposure to other people outside this building” (S11).

Participants also described how the presence of rapid testing improved the shelter's reputation among external stakeholders, including volunteers and donors. “When I've told people, whether they're donors or visitors, or volunteers, that, ‘oh, yes, we can do rapid testing’—like that's a relief to them” (S2). Others emphasized how testing signaled organizational responsibility: “Perceived by the general community as like, proactive and responsible steps” (S2).

However, the shelter's role in protecting community health was constrained by a lack of enforcement authority. While staff could recommend isolation, they could not prevent guests from leaving the facility while infectious. “Because I don't want to keep them out in public. But at the same time, like I don't have the power to keep them back in respite” (S10).

In the early stages of the pandemic, staff had positive perceptions of community support, particularly from the HD who played a central role in guidance and facilitating isolation. “they transported people to isolation sites early on, they helped with hotels, they helped get food to people that were in hotels … because that was something that we were not able to do.” (S10) In some cases they found that working to coordinate COVID-19 mitigation strategies strengthened existing partnerships: “Because we've had such consistent communication our teams have had to work together to coordinate. I think it strengthened our relationship with them. That's been a nice little side effect.” (S13). Other community entities beyond immediate partners also offered occasional support, for example in coordinating the annex: “one of the churches volunteered to be that site. So that kind of helped with the people that were not able to come in [during lockdowns], they at least had somewhere to go …” (S10).

As the pandemic progressed however, partnerships became strained, particularly in relation to isolating cases. Initially, local hotels, under contract with the HD, offered discounts on their empty rooms from the state shelter-at-home and social distancing orders. However, as the public reopened, these arrangements began to collapse; “[their] want to help us kind of dwindled away.” (S6) Staff participants identified compounding factors to this change which were primarily the reduced HD funding and occasional behavioral incidents that made hotels reluctant and ultimately unwilling to work with the HD or shelter, “we were able to use hotels initially, but our population is not a terribly compliant population. And we had people who we had placed as COVID patients who wouldn't wear their mask or roaming around the hotels, some of them caused damages. The health department had very similar issues, so we couldn't use them all the time” (S13) And once HD funding ended, “still no place, there's no funding for COVID positive folks …” (S12) leaving the shelter to either cover hotel costs or use its limited medical respite beds.

In the absence of hotels and HD funding, shelter administration felt the community could've done more when isolating positive cases became their sole responsibility, as one staff member stated: “We're always where everything lands … we wish the whole community would help more. But that's a bigger issue right? Now, it always lands here. So we're used to it.” (S13).

#### 2.1.3. Conclusion of on-site rapid testing

Despite initial enthusiasm and perceived benefits, the shelter ulti-mately ceased on-site rapid testing due to persistent space and infrastructure constraints. As COVID-19 waves continued, it became increasingly clear that the shelter could not sustainably operate as an isolation facility. This institutional reckoning was most clearly articulated by a member of shelter leadership who wrote:

“I believe we have to get out of the contagious illness isolation business. (Long-term we should consider how the space flows and really consider some structural changes.) There is no other space we are going to use for isolation in the building. We just don’t have an appropriate space. We weren’t ever intended to be an isolation ward.”

(S13)

This statement marked a turning point, prompting consultation with the local health department. In a letter dated January 9, 2023, the county health officer concluded:

“In my medical opinion, COVID-19 testing no longer needs to happen at the center. Patients who present with symptoms or have tested positive do not need to be isolated but shall wear masks as much as possible.”

Following this decision, the shelter formally discontinued rapid testing.

## 3. Discussion

Drawing on shelter staff and guest interviews alongside a contextual timeline of federal, state, county, and organizational policies, this study explored both the benefits and unintended consequences of implementing rapid testing for COVID-19 in a homeless shelter setting. In doing so, this work situates shelter-level experiences within a broader socio-political context, illustrating how local homelessness service providers were forced to navigate overlapping crises of housing insecurity and public health during a pandemic without sufficient infrastructure or policy support.

Previous research has demonstrated the promise of rapid testing for COVID-19 in congregate settings, with studies documenting effective outbreak control through early detection and confirming that the process of conducting testing was operationally feasible in shelter settings (Aranda-Díaz et al., 2022; Ogbonna et al., 2024; Rodriguez, Cromer, et al., 2022; Scott et al., 2022). One Boston-based multi-agency response reported higher positivity rates when transitioning from symptom screening to universal testing (Baggett, Racine, et al., 2020), while a pilot testing event at a congregate shelter in Denver demonstrated the value of coordinated interdisciplinary approaches (Scott et al., 2022). However, these studies consistently highlight that successful implementation of testing programs required substantial resources, including increased staffing, expanded isolation capacity, and sustained external support (Aranda-Díaz et al., 2022). While resource requirements were significant, particularly for universal or frequent testing programs, the improved outbreak control and reduced transmission observed in several studies suggested these investments were worthwhile for maintaining shelter operations during the pandemic, a priority emphasized in our work (Ogbonna et al., 2024). At the same time, the initiatives in these studies were short-term, lasting at most a few months, and took place in 2020-early 2021, therefore they do not fully assess the challenges of sustainability throughout the pandemic. Most also relied on external or temporary facilities for isolation as alternative sites that shifted the physical and organizational burden away from homeless shelters.

This study offers a longitudinal perspective of a shelter's experience beyond the initial crisis phase, documenting the operational reality as community support evolved, funding diminished or was withdrawn, and new variants and challenges emerged. We found that while rapid testing addressed urgent access barriers and provided both staff and gusts with a critical tool, the sustainability of testing depended on the infrastructure available to manage its consequences of isolation and quarantine. Our longitudinal data reveal the operational and ethical strain of deploying a long-term public health intervention without adequately sustained infrastructure. A London-based study reported a similar experience, where hotels effectively accommodated isolation until capacity changed and emergency funding ended, after which PEH filtered back into hostels causing increased outbreaks (Ogbonna et al., 2024). Our findings similarly demonstrate how the initial promise of shelter-based testing gave way to unsustainable burdens once external support receded.

Across all socioecological levels, we identified key benefits that aligned with prior research while revealing impacts not fully captured in earlier studies. At the individual level, testing addressed many well documented logistical barriers for PEH including mobility, transportation, and awareness challenges (Johannesson et al., 2023; Rodriguez, Martinez, et al., 2022). Interpersonally, it offered reassurance in a high-risk environment to both guests and staff by strengthening the perception that cases were being identified and addressed. Notably, our data revealed that guests' willingness to participate in testing extended beyond personal protection to encompass community concern. This reflects a shared commitment to reducing transmission and demonstrates the capacity of structurally vulnerable populations to engage in collective health protection when trusted interventions are available, an important consideration when developing public health strategies to engage this population in infectious disease control (Ziolkowski et al., 2024). At the institutional level, testing provided the shelter with greater autonomy over operational decision-making and outbreak control. At the community level, shelter-based testing helped limit community transmission and garnered positive community perceptions of the shelter's organizational responsibility.

Yet these benefits were met with challenges across all socioecological levels, which intensified as the pandemic progressed and external supports diminished. While shelter-based rapid testing improved access to COVID-19 screening, it also created significant challenges, as homeless shelters are not typically designed to accommodate proper isolation or medical care for individuals who test positive. At the individual level, isolation in inadequate shelter spaces restricted access to hygiene and other care needs, and lockdowns meant no access to the shelter at all for new or past PEH during outbreak windows. Guest experiences during isolation revealed not only physical constraints but also the affective and psychological toll of confinement in spaces not designed for extended occupancy. This discomfort reflected the fundamental structural mismatch between isolation requirements and the shelter's operational capacity. Interpersonally, inconsistent communication about how positive cases were managed led to privacy concerns and mistrust, while onsite isolation created emotional burdens for some. The tension experienced at this level, in particular the moral distress experienced by staff, relates directly to a growing body of literature on emotional labor in social service settings, which Hochschild (1984) describes as the internal effort needed to manage one's own feelings in order to meet organizational requirements (ARussell, 2012). Kerman et al.‘s (2022) study on ‘systems trauma’ argues this labor is amplified when homelessness service providers are forced to navigate ethical contradictions while operating in under-resourced systems. Our findings demonstrate how the pandemic exacerbated this burden as staff primarily accustomed to care providing roles had to act as public health enforcers (Kerman et al., 2022). Institutionally, lockdowns and limited isolation space created new challenges that strained the shelter's core mission and low-barrier philosophy, creating moral and logistical challenges for staff. This was also felt by leadership, leading to inconsistent enforcement of polices. Leadership also had the added concern of lower intake performance metrics for the shelter during lockdown periods. At the community level the decline of hotel isolation and local health department capacity shifted the full burden of outbreak mitigation onto the shelter, which was never designed to function as an isolation facility.

These multi-level challenges were not solely isolated within each socioecological level, rather, they reflected underlying decisions across federal, state, local, and organizational levels. Federal agencies established emergency declarations, approved tests, and distributed supplies, but provided limited guidance for homelessness service providers and uneven support for housing stability. The state government enacted stay-at-home orders, mask mandates, and reopening phases, which directly influenced shelter operations. The county health department, in turn, dictated lockdowns and initially facilitated hotel-based isolation before resource constraints curtailed these options. Within this framework, the shelter functioned both as a social service provider and as an informal public health authority, a role it neither sought nor was equipped to sustain. This multi-level dynamic highlights how state power is enacted through public health policies that unevenly distribute responsibility, exemplifying what Farmer (1996) termed “structural violence”, where systemic choices harm individuals by preventing access to the basic resources and rights necessary to protect their own health (Farmer, 2009). As Jenkinson et al. (2024) observed in their study of COVID-19 among PEH, this violence is experienced where policy mandates and individual or interpersonal interactions converge, not simply the result of ‘proximal’ individual behaviors or ‘distal’ structures (Jenkinson et al., 2025). Our analysis shows how this convergence played out within the organization. In this case, structural inequities left a homelessness services agency compensating for gaps in public health infrastructure. The state shifted the primary responsibility for disease management onto a precarious social service sector. In doing so, the health and safety of a marginalized population was treated as a logistical problem for the shelter to solve, rather than a fundamental public health obligation for the community. The shelter's experience illustrates how emergency responses devolve onto local organizations when higher-level supports recede, exacerbating inequities already borne by structurally vulnerable populations.

The shelter's decision to discontinue isolation and testing in January 2023 reflects a broader reckoning about sustainability. Having once assumed responsibilities for disease control, shelter leadership explicitly rejected the role of operating as a medical isolation facility. This trajectory also shaped future responses as staff later preemptively decided not to isolate for Mpox (Rodriguez et al., 2024). These decisions reflect not a failure of will or commitment, but a necessary boundary-setting after months of managing contradictions that the organization's design and mission could not sustainably accommodate. In the context of crisis governance, where emergency responsibilities proliferate and resources contract, the shelter's strategic boundary-setting decisions represent a deliberate prioritization of its core mission over unsustainable public health functions that had exceeded organizational capacity. These findings raise critical questions about who bears responsibility for protecting vulnerable populations in public health crises. Shelters are essential community organizations, but they cannot sustainably act as isolation wards or public health authorities without infrastructure, policy clarity, and resource support. As prior studies in Boston, Portland, and London suggest, successful testing interventions required strong multi-level coordination and dedicated facilities (Baggett, Racine, et al., 2020; Ngandu et al., 2024; Ogbonna et al., 2024).

Overall, our findings support and underscore numerous studies demonstrating that testing availability alone is insufficient. Without parallel investments in isolation infrastructure, policy coordination across government levels, and sustained community support, homeless shelters risk being burdened with responsibilities that exceed their mission, capacity, and resources. These findings have critical implications for pandemic preparedness and for how society responds to health crises among structurally vulnerable populations.

Future preparedness should focus on building and maintaining cross-sector partnerships, which proved integral to making rapid testing accessible for underserved populations. As funding and resources can be precarious, developing community networks and committees offers a valuable mechanism for creative problem-solving during crises. For example, our team's weekly committee formed by academic partners, the homelessness services organization, and other stakeholders during the pandemic provided a channel for disseminating testing findings and mobilizing resources such as masks and cleaning supplies, even though it could not fully resolve the challenge of isolation space.

In addition, federal and state emergency policies should embed flexibility so that local service providers can adapt without bearing disproportionate burdens. Physical shelter design must also incorporate outbreak preparedness, including dedicated isolation areas that do not compromise sheltering and rehousing functions. Leveraging community committees can help address capacity limitations in existing facilities, but long-term solutions require structural changes. Finally, interventions should center the expertise of people experiencing homelessness (PEH), recognizing their dual concern for self-protection and community wellbeing. Their inclusion is essential for developing responses that are both effective and equitable.

This study has several limitations. First, as a qualitative case study of a single organization in a Midwest city, the findings may not be generalizable to all homelessness service settings. Recruitment was limited to participants who participated in shelter-based testing, which might exclude the perspective of guests who choose not to test and could have meant a higher rate of positive perceptions of testing among guest participants.

## 4. Conclusion

The implementation of COVID-19 rapid testing in a Midwestern homeless shelter addressed urgent access barriers and provided staff and guests with a critical tool during the pandemic. However, in the absence of adequate infrastructure and sustained policy support, testing also created dilemmas that strained shelter capacity and diverted attention from core missions. The shelter became a reluctant public health actor, tasked with managing disease while simultaneously confronting the broader crisis of homelessness. This study underscores that emergency responses cannot be sustained by frontline service agencies alone. Effective and equitable public health strategies for structurally vulnerable populations require sustainable, multi-level commitments that extend beyond an immediate crisis.
